# Regulation Effect of a Chinese Herbal Formula on Flora and Mucosal Immune Secretory Immunoglobulin A in Rats

**DOI:** 10.1155/2018/4821821

**Published:** 2018-11-08

**Authors:** Tian-hao Liu, Xiao-mei Zhang, Ni-ping Han, Yang Liu, Yue-ying Wu, Xiao-ya Li, Zhong-shan Yang, Jia-li Yuan

**Affiliations:** ^1^School of Basic Medical Sciences, Yunnan University of Traditional Chinese Medicine, Yuhua Road, No. 1024, Kunming, Yunnan 650500, China; ^2^College of Traditional Chinese Medicine, Jinan University, No. 601 West of Whampo Avenue, Guangzhou, Guangdong 510632, China

## Abstract

Flora and mucosal immunity are considered to be the barrier, which is associated with multiple respiratory diseases, including recurrent respiratory tract infection (RRTI). Fei-Xi-Tiao-Zhi-Fang (FTF) is a traditional Chinese herbal formula used in the treatment of RRTI. However, the mechanism is little known. This study aims to identify the function of FTF in flora and mucosal immune secretory immunoglobulin A (sIgA) in the model of RRTI rats. The samples of intestine and lung were collected to detect sIgA, short chain fatty acids (SCFAS), and flora with enzyme-linked immunosorbent assay (ELISA), gas chromatography, and 16S rDNA sequencing. The body weight and viscera index were increased dynamically in RRTI rats after the administration of FTF. Furthermore, the types and proportions of aboriginal flora were significantly changed in the model group, whereas the altered flora was rescued in the FTF administration group.* Desulfovibrio* increased in the intestinal microflora and* Ralstonia *and* Blautia* decreased in the pulmonary microflora at the genus level, similar to that in the normal group. In addition, the expressions of sIgA in pulmonary and intestinal tissues were significantly upregulated and the level of SCFAS was increased in FTF group compared to the RRTI model group. Our study suggests that FTF can alleviate the symptoms of RRTI by increasing sIgA and SCFAS, recovering flora, and improving the immunity.

## 1. Introduction

Recurrent respiratory tract infection (RRTI) is considered to be a chronic respiratory disease, which is characterized by flora infection and immune dysregulation. The disease is manifested in the respiratory tract and intestine and the changes of intestinal flora can also affect respiratory diseases [1–3]. According to the theory in traditional Chinese medicine, the relationship between the lung and the large intestine is the interior-exterior corresponding relationship and the lung and intestine axis have confirmed the obvious connection between the gut and the respiratory tract [4–6]. Many scholars have also explained it in many aspects. We can prevent respiratory diseases by changing the intestinal microenvironment, adjusting the respiratory environment, and affecting intestinal functions. Therefore, according to the famous ancient Chinese medicine decoction “Yupingfengsan” and “Cangerzisan” (both are important decoction for treating respiratory diseases), Chinese medicine experts created FTF, which could change the intestinal microenvironment and adjust the respiratory tract infection [7–9]. When FTF is used in clinic, it has an obvious curative effect and can alleviate the respiratory tract infection and intestinal symptoms.

Flora is closely related to mucosal immunity. The bacteria can act on the mucous membrane, which can also affect the flora. Intestinal flora can metabolize short chain fatty acids (SCFAS), which can act on the intestinal mucosa, thereby affecting the body [10, 11]. Pulmonary and intestinal mucosae are attached to a large number of bacteria and the microflora and immunity of lung and intestine may be considered as the biological basis of the lung-intestinal correlation. The changes in the composition and structure of bacterial flora can be considered as the changes in the bacterial flora of the lung and intestine and the change in mucosal secretory immunoglobulin A (sIgA) may represent the level of mucosal immunity in the lung and intestine [12]. All the results suggest that FTF may play a role in the prevention and treatment of RRTI by regulating the flora and mucosal immunity. However, the direct evidence demonstrating the mechanism of FTF in the prevention and treatment of RRTI was seldom reported. In this study, we observed the changes in the flora and immunity in the lung and intestine of rats in order to illustrate the underlying mechanisms and provide scientific evidences for the role of FTF in the prevention and treatment of RRTI.

## 2. Materials and Methods

### 2.1. Animals

A total of 63 SPF Wistar rats (130±10 g, 3-4 weeks old, male) were purchased from Chengdu Dashuo Experimental Animal Co., Ltd. The rats were housed in standard environmental conditions and could freely access to diet and water. All groups were fed adaptively for seven days before the study. The study was approved by Committee on Animal Experimental Ethics of Yunnan University of Traditional Chinese Medicine and all the experimental methods were performed in accordance with the relevant guidelines and regulations.

### 2.2. Grouping

The animals were randomly divided into seven groups: normal control group (K), model control group (MX), high-dose FTF group (HZ), medium-dose FTF group (MZ), low-dose FTF group (LZ), FTF atomization group (AD), and positive medicinal control group (YZ) and each group contained 9 rats.

### 2.3. Immunosuppression and Dysbacteriosis in Rats and the Treatments

The study was divided into four phases. From the first to the sixth day, the rats were fed adaptively. From the seventh to fourteenth day, the rats were treated with the mixture of antibiotics and hormone every 24 h. From the fifteenth to the twenty-first day, the rats were treated with different drugs every 24 h. All the rats were killed on the twenty-second day.

### 2.4. Construction of the Model

Cefradine capsules (Shijiazhuang Pharmaceutical Group Ouyi Pharma Co., Ltd.), gentamycin sulfate (Shanghai Shenguang Company), and dexamethasone sodium phosphate injection (Shanxi, Ruicheng Kelon Veterinary Medicine Co., Ltd.) were combined to obtain a mixture of 22.6 g/L in the proportion of 1:5:6, which was administered intraperitoneally to rats (2 mL per day). From the seventh day, the mixed suspension was injected to the groups for 8 consecutive days except the normal control group once a day. Meanwhile, normal saline was injected to the normal control group in the same way.

### 2.5. Preparation of FTF

The FTF was prepared by Yunnan Hongxiang Yixintang Pharmaceutical Limited Ltd. FTF herbs include huangqi (astragalus), fangfeng (windbreak), baizhi (angelica root), cangerzi (cocklebur), xinyihua (magnolia flower), xingren (almond), tinglizi, and gancao (licorice). Firstly, after adding 800 mL of deionized water, FTF herbs were soaked for 1 h and then decocted over a low flame for 40 min after boiling. Then the filtrate was collected with gauze. Secondly, 600 mL of deionized water was added to the dregs of a decoction and then decocted in the same way. Then the filtrate was collected with gauze. Thirdly, 600 mL of deionized water was added to the dregs of a decoction and then decocted in the same way. Then the filtrate was collected with gauze. Finally, all filtrates were combined together, made into extractum, and stored at 4°C. The dose of FTF for rats was calculated with body surface area. The medium dose was the equivalent dose (0.5 g/mL) and the ratio of low, medium, and high doses was 1:2:4 [13].

### 2.6. Chemical Composition Analysis of FTF Extract

The FTF extract was diluted by 10 times. Then 1 mL of diluted FTF extract was added into a 2-mL centrifuge tube, centrifuged at 13,000 r/min for 10 min, filtered through 0.22-*μ*m microporous membrane, and then loaded into a 1.5-mL automatic sampling bottle to obtain the covering liquid sample. Blank control samples were obtained under the same conditions. The samples were stored in the refrigerator at 4°C for analysis and the storage time should not exceed 24 h. The active components of FTF extract were analyzed qualitatively by ultrahigh performance liquid chromatography-time-of-flight high resolution mass spectrometry. The 20 components in the FTF extract with the response value greater than 100,000 are shown in Table 1.

### 2.7. Treatment Drugs

From the fifteenth to the twenty-first day, the rats in the HZ, MZ, and LZ groups were fed with different doses of FTF decoction orally every 24 h and the AD group was fed with atomized decoction with the equivalent dose every 24 h. Meanwhile, the YZ group was treated with Broncho-Vaxom with the equivalent dose and the MX and K groups were treated with the same amount of normal saline in the same way.

### 2.8. Index Detection

On the twenty-second day, all rats were sacrificed and tissue samples were collected under aseptic conditions.

### 2.9. Viscera Index

The thymus and spleen of all rats were collected and weighed and the viscera indexes were calculated according to the formula (viscera index = organ mass (mg) / body weight (g) ∗ 100%).

### 2.10. Enzyme-Linked Immunosorbent Assay (ELISA) for the Quantitative Detection of sIgA

The pulmonary and intestinal tissues of all groups were obtained. The grinding bowl was precooled with liquid nitrogen. Then, 400 mg of the tissue was put into the grinding bowl. Liquid nitrogen was added for grinding. The crushed tissue was poured into EP tube and 1 mL of phosphate buffer solution (pH 7.4) was added and centrifuged for 20 min with a cryogenic centrifuge at 3000 rpm. Finally, the expression of sIgA was detected according to the instructions of ELISA kit supplied by Nanjing Senbeijia Biological Technology Co., Ltd. (Nanjing, China).

### 2.11. High-Throughput Sequencing

The intestinal contents and lung tissues were taken under aseptic conditions and stored at -80°C. Three samples were randomly selected from each group of lung tissues and intestinal components (lung: FK, FMX, FMZ, FAD, FYZ; intestine: CK, CMX, CMZ, CAD, CYZ) for the sequence analysis of flora. The next-generation sequencing library preparations, Illumina MiSeq sequencing, and V3 and V4 hypervariable regions of 16S rDNA were provided by GENEWIZ, Inc. (Suzhou, China).

### 2.12. Expression of SCFAS Tested by Gas Chromatography

Three samples were randomly selected with 3 intestinal components of each group (K group, MX group, MZ group, AD group, and YZ group). After adding 2-mL of water, the samples (1 g) were homogenized for 2 min. Then 1 mL of ether was added for 10-min extraction and then centrifuged at 4000 rpm for 20 min. Then 1 mL of ether was added for 10-min extraction and then centrifuged at 4000 rpm for 20 min. The two extracts were combined and volatilized to the volume of less than 1 mL. Then 0.1 mL of 1000 mg/L ether internal standard solution was added and the volume was fixed to 1 mL. Further, the intermixture was transferred to be tested. SCFAS, including acetic acid (CAA), propionic acid (CPA), isobutyric acid (CIA), ethacetic acid (CEA), common valeric acid (CCVA), and pentanoic acid (CPEA), were tested by gas chromatography according to the operation process (GCMS ISQ LT). The experimental materials required in this section were provided by Qingdao Yixin Testing Technology Service Co., Ltd.

### 2.13. Statistical Analysis

The 16S rDNA data analysis was performed with the QIIME package data and R programming language. Other results were presented as mean ± SD. The experimental data were analyzed by SPSS statistical software and GraphPad Prism 6.* P*<0.05 or* P*<0.01 was considered to be statistically significant or extremely significant.

## 3. Results

### 3.1. Weight Changes

**Figure 1:**
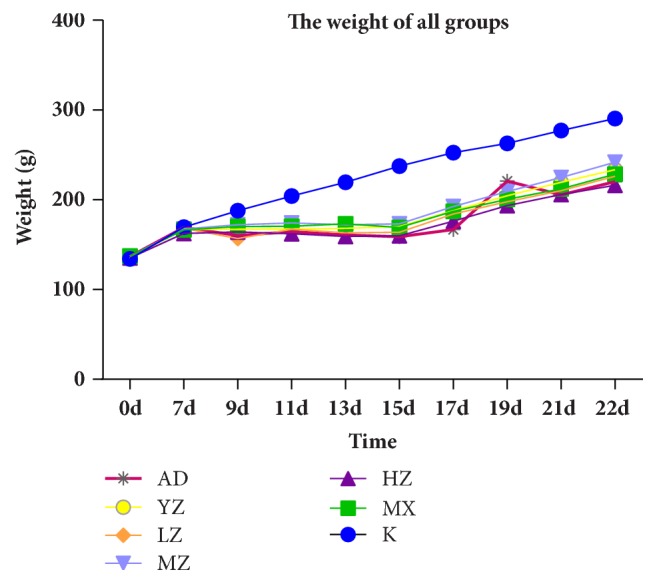
**Changes in the weight of rats during the experiment. **From Day 0 to Day 7, rats were naturally fed; from Day 8 to Day 15, rats were modeled; from Day 15 to Day 22, rats were treated. In the modeling process, the weight of the rats in the K group was significantly higher than that of the MX group; when the treatment was given, the MZ group rose significantly and the weight change of the AD group was tremendous at the beginning of the treatment.

In the seven days of adaptive feeding, the weight of all groups increased significantly. From Day 7 to Day 14, the weight of all the rats except the rats in the K group remained almost unchanged. From Day 14 to Day 21, the weights of the rats in the MZ and YZ group were higher than those in other groups except the K group. Meanwhile, the weight of the AD group increased obviously in the first several days (Figure 1).

### 3.2. FTF Dynamically Increased the Viscera Index

**Figure 2:**
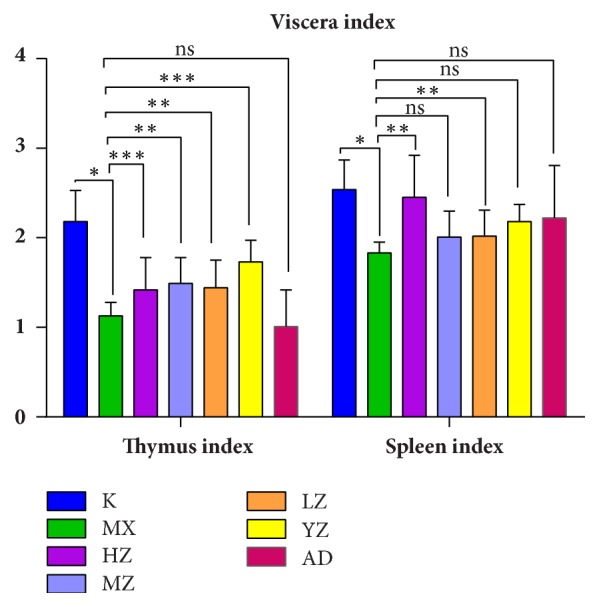
**FTF dynamically regulated viscera index. **The left side is the thymus index of each group (*n*=8) and the right is the spleen index of each group (*n*=8). Data are presented as mean ± SD. ^∗^*P<0.05* versus the K group; ^∗∗^*P<0.05 *and ^∗∗∗^*P<0.01 *versus the MX group.

Compared with the K group, the thymus index of the MX group decreased significantly (*P*<0.05). Compared with the MX group, the thymus index of the HZ, MZ, and LZ groups significantly increased (*P*<0.05) and the thymus index of the AD group decreased slightly by aerosol (*P*>0.05), YZ>MZ>LZ>HZ>AD. There was no significant difference among the groups (*P*>0.05). Compared with the K group, the spleen index in the MX group rats decreased significantly (*P*<0.05); compared with the MX group, the spleen index of the HZ group significantly increased (*P*<0.05); the spleen index of the MZ and LZ group increased (*P*>0.05). There was no significant difference among the groups (*P*>0.05), HZ>AD>YZ>LZ>MZ (Figure 2).

### 3.3. FTF Significantly Improved the Expression of sIgA in Pulmonary and Intestinal Tissues

**Figure 3:**
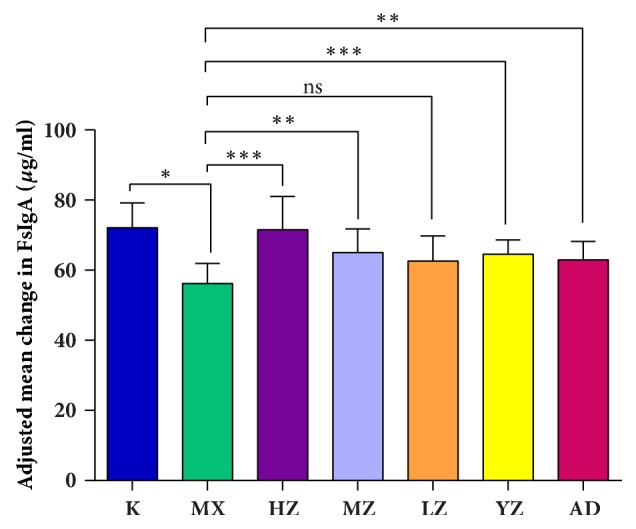
**FTF significantly improved the expression of sIgA in pulmonary tissues. **Data (*n*=8) are presented as mean ± SD. ^∗^*P<0.05* versus the K group; ^∗∗^*P<0.05 *and ^∗∗∗^*P<0.01 *versus the MX group.

Compared with the K group, the sIgA level in lung tissues of the MX group rats decreased significantly; compared with the MX group, the sIgA levels in lung tissues of the HZ group rats increased significantly (*P*<0.01) and the sIgA levels in lung tissues of MZ, YZ, and AD groups increased (*P*<0.05). The sIgA levels in the LZ group rats increased significantly (*P*>0.05), HZ>MZ>YZ>AD>LZ (*P*>0.05) (Figure 3).

**Figure 4:**
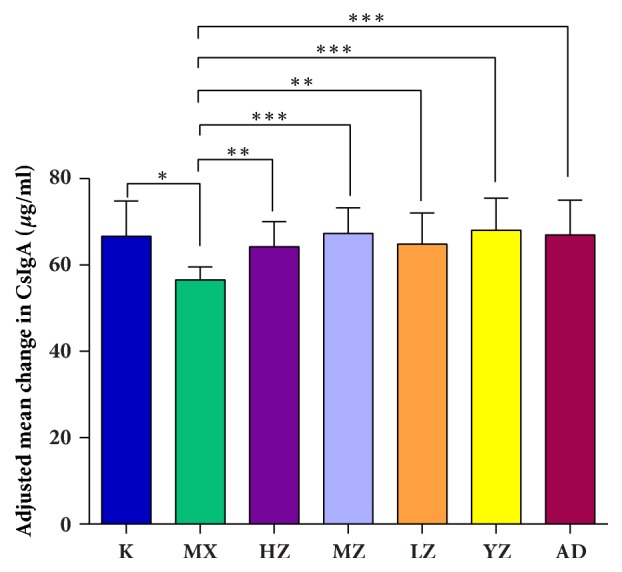
**FTF significantly improved the expression of sIgA in intestinal tissues. **Data (*n*=8) are presented as mean ± SD. ^∗^*P<0.05* versus the K group; ^∗∗^*P<0.05 *and ^∗∗∗^*P<0.01 *versus the MX group.

Compared with the K group, the sIgA level in intestinal tissues of the MX group decreased significantly; compared with the MX group, the sIgA levels in lung tissues of the MZ, YZ, and AD groups increased significantly (*P*<0.01) and the HZ and LZ groups increased (*P*<0.05), YZ>MZ>AD>LZ>HZ. There was no significant difference among the groups (*P*>0.05) (Figure 4).

### 3.4. FTF Significantly Raised the Expression of SCFAS

**Figure 5:**
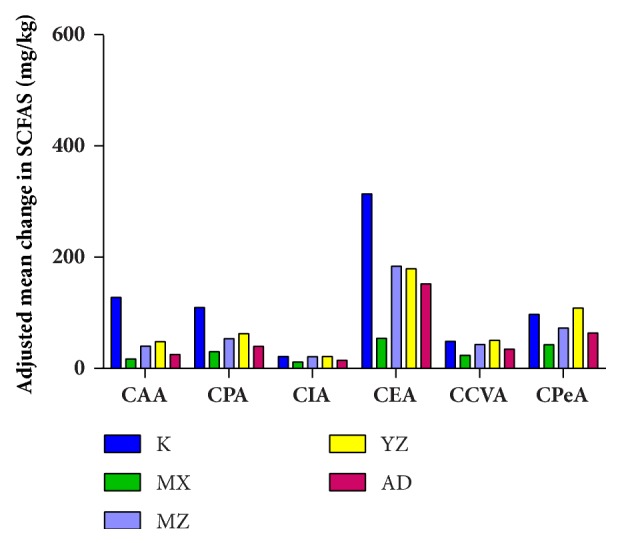
**Expression of SCFAS in rats of every group. **SCFAS, including acetic acid (CAA), propionic acid (CPA), isobutyric acid (CIA), ethacetic acid (CEA), common valeric acid (CCVA), and pentanoic acid (CPEA) were detected in every group (*n*=3).

SCFAS including cetic acid (CAA), propionic acid (CPA), isobutyric acid (CIA), ethacetic acid (CEA), common valeric acid (CCVA), and pentanoic acid (CPEA) were detected. All these acids in the MX group decreased compared with the K group. The SCFAS in MZ and AD groups was significantly increased compared with the MX group (Figure 5).

### 3.5. FTF Significantly Regulated the Compositions and Structures of the Pulmonary and Intestinal Flora

**Figure 6:**
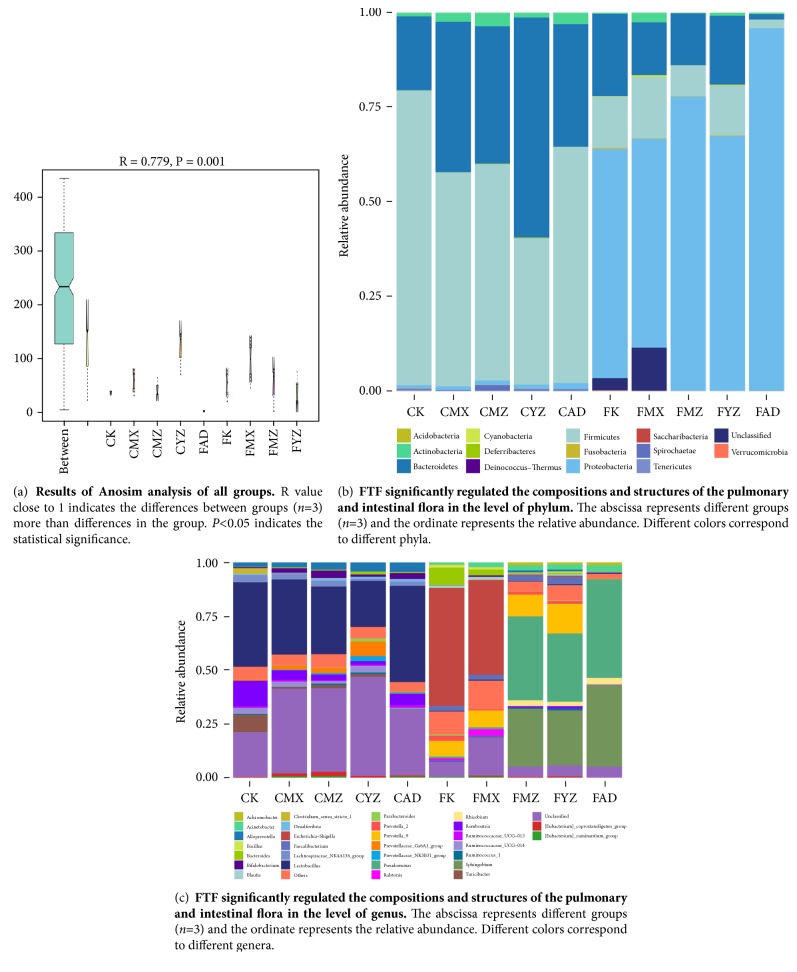


The result of 16S rDNA gene sequence (Figure 6(a)) indicated that there was a significant difference (*R *= 0.779,* P *= 0.001) among the groups. The changes in the phylum level showed that the intestinal flora in the K group was mainly composed of* Firmicutes*,* Bacteroidetes*,* Actinobacteria*,* Proteobacteria*, and* Spirochaetae*. Compared with the K group, the intestinal microflora in the rats of the MX group showed the decreased levels of* Firmicutes*,* Proteobacteria,* and* Spirochaetae* and the increased levels of* Bacteroidetes *and* Actinobacteria*. Compared with the MX group, the MZ group showed the increased levels of* Firmicutes*,* Spirochaetae,* and* Actinobacteria*, the decreased level of* Bacteroidetes*, and the unchanged level of* Proteobacteria*; the AD group showed the increased levels of* Firmicutes*,* Actinobacteria,* and* Proteobacteria*, the decreased level of* Bacteroidetes*, and the unchanged level of* Spirochaetae*; the YZ group showed the decreased levels of* Firmicutes* and* Actinobacteria* and the increased levels of* Proteobacteria*,* Spirochaetae,* and* Bacteroidetes*. Meanwhile, the pulmonary flora in K group was mainly composed of* Proteobacteria*,* Bacteroidetes*,* Firmicutes*, and* Actinobacteria*. The MX group showed the decreased levels of* Proteobacteria* and* Bacteroidetes *and the increased levels of* Firmicutes* and* Actinobacteria* compared with the K group. After the rats of the MZ, AD, and YZ groups were treated, the MZ group showed the increased levels of* Proteobacteria* and* Bacteroidetes* and the decreased levels of* Firmicutes* and* Actinobacteria*; the AD group showed the increased level of* Proteobacteria* and the decreased levels of* Bacteroidetes*,* Firmicutes, *and* Actinobacteria*; the YZ group showed the increased levels of* Proteobacteria *and* Bacteroidetes *and the decreased levels of* Firmicutes* and* Actinobacteria *(Figure 6(b)).

At the genus level, the intestinal microflora of rats was composed of* Bifidobacterium, Alloprevotella, Prevotellaceae_Ga6A1_group, Ruminococcaceae_UCG, Lachnospiraceae_NK4A136_group, Turicibacter-014, Romboutsia, Ruminococcus_1, [Eubacterium]_coprostanoligenes_group, Desulfovibrio, Ruminococcaceae_UCG, [Eubacterium]_ruminantium_group, Parabacteroides-013, Prevotellaceae_NK3B31_group,* and* Lactobacillus*.

The pulmonary flora was composed of* Pseudomonas, Sphingobium, Clostridium_sensu_stricto_1, Faecalibacterium, Prevotella_2, Achromobacter, Acinetobacter, Rhizobium, Ralstonia, Bacillus, Blautia, Escherichia* and* Shigella, Prevotella_9*, and* Bacteroides* (Figure 6(c)). Obviously, all these evidences showed that FTF could significantly regulate the compositions and structures of the pulmonary and intestinal florae.

### 3.6. Correlation Analysis of Flora and Mucosal Immune sIgA in Rats

**Figure 7:**
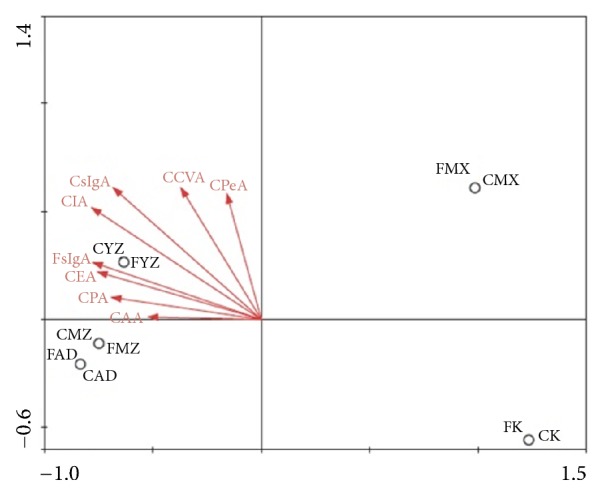
**Results of RDA/CCA analysis. **RDA/CCA is a ranking method based on correspondence analysis, which is mainly used to reflect the relationship between flora and environmental factors. The angles between environmental factors indicate positive and negative correlation between environmental factors (acute angle: positive correlation; obtuse angle: negative correlation; right angle: no correlation). As indicated by the vertical projection points of different samples on various environmental factors, the closer the distance between the vertical projection points is, the more similar the environmental factor among different samples is, indicating that the degrees of the influences of the environment factor on different samples are similar.

**Figure 8:**
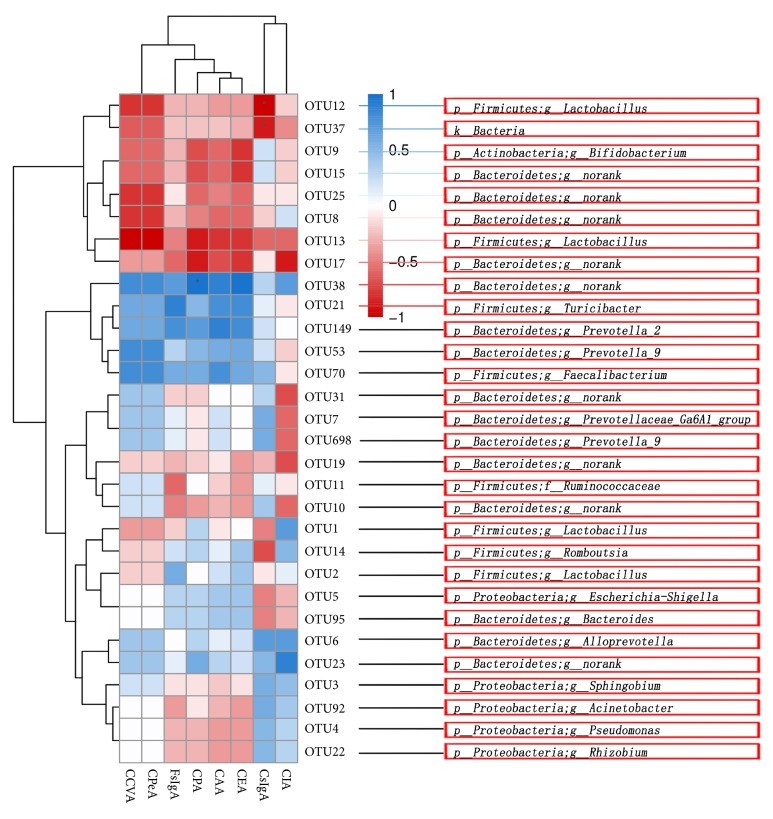
**Relationship between the environmental factors and community composition was intuitively displayed by Spearman using R language. **The data were based on OTU abundance or species richness and environmental factor data. The correlation coefficient r [-1~1], r>0 were positively correlated, and r<0 was negatively correlated. In significance tests,* P *values between 0.01 and 0.001 were marked as ∗ ∗;* P *values between 0.01 and 0.05 were marked as ∗.

According to the correlation analysis result, the six parts of SCFAS were positively correlated with sIgA of lung and intestine. At the same time, there was a significant positive correlation between sIgA of the lung and the intestine (Figure 7). After the analysis, we identified the positive and negative correlations of the first 30 OTUs between SCFAS and sIgA (Figure 8).

## 4. Discussion

Flora and immunity are important components of the body and play an important role in anti-inflammatory defense against pathogens. Traditional Chinese medicine (TCM) has a long history and rich theory. A variety of useful chemical components have also been detected from Chinese medicine [14–16]. Many diseases have been cured with TCM nowadays in China, but many unknown mechanisms have restricted its widespread transmission in the world. Traditional Chinese medicine theories such as “lung intestine axis” and “the corresponding relationship between lung and large intestine” show that the respiratory tract is closely related to the intestinal tract. In our study, when the rats were stimulated by antibiotics and hormone, they were in a state of flora imbalance and immunosuppression. To our knowledge, this study was the first time to establish the model of immunosuppression and dysbacteriosis in rats on the basis of the immunosuppression and dysbacteriosis rats [17–19].

Viscera index is considered to represent the level of immune development [20–22]. sIgA can represent the level of mucosal immunity [23, 24]. The interaction between flora and sIgA is significant [25]. SCFAS are confirmed to be the metabolites of intestinal flora, which also interacts with the gut [26, 27]. The changes in viscera index, the expressions of sIgA and SCFAS, and the changes in the pulmonary and intestinal flora in our study indicated that FTF could regulate the flora and mucosal immunity of the lung and intestine. The flora and mucosal immunity may be the scientific connotation of the lung-intestine correlation.

Aerosol inhalation of medicine has a significant effect on respiratory diseases [28, 29]. This study designed the AD group and found that atomized FTF controlled the disease quickly as indicated by the change in weight. The study may provide some ideas for the development of TCM atomization agent. Oral administration of FTF and aerosolized FTF both affect the lung and intestine simultaneously. Additionally, this study revealed that the specific bacterial community was associated with SCFAS and sIgA. We found that* g__Lactobacillus* was negatively correlated with intestinal mucosal sIgA, suggesting that* g__Lactobacillus *might inhibit intestinal mucosal immunity. The detailed mechanism between* g__Lactobacillus *and sIgA needs further study.

In conclusion, this study suggests that FTF can regulate the sIgA and the composition and structure of flora in the lung and intestine and increase SCFAS in intestinal microbiota metabolism. In addition, it reveals the possible biological basis of the connection of lung and intestine. Although the specific mechanisms that FTF directly acts on these indicators, as well as some specific bacterial communities and immune sIgA, are not clear, this study suggests a potential direction for the next research.

## Figures and Tables

**Table 1:** 20 components in FTF extracts.

Components	Formula	Adduct	Found Mass	Adduct	RT	Intensity
Glycyrrhizic Acid	C42H62O16	-H	821.39266	-4.7	20.06	1555680
4'-O-beta-Glucopyranosyl-5-O-MethylviSamminol	C22H28O10	+H	453.17675	2.7	11.01	725986
Prim-O-glucosylcimifugin	C22H28O11	+H	469.17136	2	8.28	716074
S)-tetrahydrocolumbamine	C20H23NO4	+H	342.17112	3.3	6.81	471691
Corlumidine	C20H23NO4	+H	342.17112	3.3	6.81	471691
Cimifugin	C16H18O6	+H	307.11846	2.8	10.04	325644
Adenosine	C10H13N5O4	+H	268.10425	0.8	1.56	266516
Catechingallate	C22H18O10	-H	441.0832	2	0.93	216855
Scopoletin	C16H18O9	-H	353.08646	-3.8	5.03	201272
Episappanol	C16H16O6	+H	305.10251	1.8	14.25	200565
Oxypeucedanin hydrate	C16H16O6	+H	305.10251	1.8	14.25	200565
Monnieriside G	C21H26O10	+H	439.16081	2.1	15.09	152817
Liquiritigenin	C15H12O4	+H	257.08138	2.1	9.35	134270
4'-methoxy Daidzein	C22H22O9	+H	431.13467	2.3	13.19	109861
Ononin	C22H22O9	+H	431.13467	2.3	13.19	109861
3'-methoxypuerarin	C22H22O10	+H	447.1297	2.5	9.15	107116
Apigenin6-glucosyl-7-o-methylether	C22H22O10	+H	447.1297	2.5	9.15	107116
Glycitin	C22H22O10	+H	447.1297	2.5	9.15	107116
Calycosin-7-glucoside	C22H22O10	+H	447.1297	2.5	9.15	107116
Trifolirhizin	C22H22O10	+H	447.1297	2.5	9.15	107116

## Data Availability

The data used to support the findings of this study are included within the article and can be made freely available. Any questions of data will be considered to be answered by the corresponding author.

## Abbreviations

RRTI: Recurrent respiratory tract infections
FTF: Fei-Xi-Tiao-Zhi-Fang
sIgA: Secretory immunoglobulin A
SCFAS: Short chain fatty acids
ELISA: Enzyme-linked immunosorbent assay
K: Normal control group
MX: Model control group
HZ: High-dose FTF group
MZ: Medium-dose FTF group
LZ: Low-dose FTF group
AD: FTF atomization group
YZ: Positive medicinal control group
CAA: Acetic acid
CPA: Propionic acid
CIA: Isobutyric acid
CEA: Ethacetic acid
CCVA: Common valeric acid
CPEA: Pentanoic acid.
